# Increasing Trends in the Pancreatitis Risk With Tumor Necrosis Factor Inhibitor Use

**DOI:** 10.7759/cureus.74245

**Published:** 2024-11-22

**Authors:** Joyce H Gu, Mark Samarneh

**Affiliations:** 1 Medicine, Lake Erie College of Osteopathic Medicine, Greensburg, USA; 2 Internal Medicine/Nephrology, St. John's Riverside Hospital, Yonkers, USA

**Keywords:** disproportionality analysis, food and drug administration adverse event reporting system (faers), medication-induced pancreatitis, pharmacovigilance, tnf alpha inhibitor

## Abstract

Tumor necrosis factor (TNF) inhibitors are a class of pharmacologic agents used to treat a wide range of immunologic diseases. We present a systematic study of the pancreatitis risk with TNF inhibitor use through a retrospective case-control design disproportionality analysis of the U.S. Food and Drug Administration Adverse Event Reporting System (FAERS) database, with data ranging from the fourth quarter of 2012 (2012 Q4) to the second quarter of 2024 (2024 Q2). Our analysis reveals an increasing trend in the pancreatitis risk with TNF inhibitors when stratified by year, with all of the TNF inhibitors studied exhibiting significant association with pancreatitis in the year 2023. Furthermore, these results hold even when controlling for sex, age, concurrent use of azathioprine, and the indication for treatment. Our findings flag the necessity for a careful investigation and monitoring of pancreatitis risk in patients undergoing TNF inhibitor therapy, especially in recent years.

## Introduction

Tumor necrosis factor alpha (TNF-α) is a key proinflammatory cytokine that plays a central role in the pathogenesis of various autoimmune conditions ranging from rheumatoid arthritis to inflammatory bowel disorders and beyond [1]. TNF inhibitors are a class of pharmacologic agents that treat autoimmune disorders by binding TNF to block its interactions. As a result, TNF inhibitors have become one of the most successful treatment therapies for a wide range of immunologic diseases [2]. TNF inhibitors are by now considered a first-line therapy for rheumatoid arthritis [3] and a mainstay in the treatment of inflammatory bowel disorders [4]. TNF inhibitors have also been found to be effective for various off-label treatments for diseases including hidradenitis suppurativa, Sjogren’s syndrome, polymyalgia rheumatica, and others [2]. The FDA has currently approved five TNF inhibitors: infliximab (IFX), adalimumab (ADA), golimumab (GOL), certolizumab (CTZ), and etanercept (ETN). IFX, ADA, GOL, and CTZ are monoclonal antibodies against TNF-α, while ETN is a fusion protein of TNF receptors with the Fc fragment of human IgG [5].

Adverse drug events are an important consideration with any pharmacologic therapy. Some of the most widely recognized adverse reactions of TNF inhibitors include infections and malignancies [6], due to the immunosuppressive effect of TNF inhibitors. However, other rarer yet more serious adverse effects of TNF inhibitors are yet to be investigated in depth. Inspired by a number of recent case reports [7-13], in this study, we investigated the association between TNF inhibitor therapy and acute and chronic pancreatitis.

Acute pancreatitis is characterized by inflammation of the pancreas along with parenchymal and peripancreatic fat necrosis [14]. The most common causes of acute pancreatitis are gallstones and ethanol abuse, but drug adverse reactions are responsible for a significant number of cases of acute pancreatitis, accounting for 0.1%-2% of acute pancreatitis incidents [14]. In particular, TNF inhibitors have been suggested to be a cause of both acute and chronic pancreatitis in a number of cases. Cinquetti et al. described a Crohn’s disease patient who developed pancreatitis upon IFX infusion [7]. Bhalla et al. and Werlang et al. both reported cases of drug-induced pancreatitis associated with IFX and ADA [8,10]. Sahu et al. reported a case of a psoriasis patient on ADA who developed pancreatitis [13]. ETN has been implicated as a cause of drug-induced pancreatitis by Miramontes et al., Gunawan et al. and Patel and Butt [9,11,12].

One important possible explanatory factor for acute pancreatitis seen in association with TNF inhibitor therapy is the concomitant use of other drugs with strong associations with acute pancreatitis. A particularly notable example is a combination therapy of TNF inhibitors with azathioprine (AZA) [15,16], which has been known for its association with drug-induced pancreatitis for decades [17,18]. For example, Cinquetti et al. suggested the concomitant use of AZA as a possible complicating factor in their case report of IFX-induced pancreatitis [7]. Thus, controlling for the use of AZA is an important consideration in the study of drug-induced acute pancreatitis due to TNF inhibitors.

## Materials and methods

Data source

In this study, we used the FDA Adverse Event Reporting System (FAERS) database as the source of data [19]. The FAERS database is a publicly available database with quarterly data on spontaneous reports of adverse drug events ranging from 2012 to 2024. Data from the fourth quarter of 2012 (2012 Q4) until the latest available data from the second quarter of 2024 (2024 Q2) were included in the study. Data were downloaded in the ASCII format from the FDA website and analyzed using the python programming language.

The FAERS database is composed of various data files that capture different aspects of adverse drug event reports. We primarily used the reaction (REAC) and drug (DRUG) files, which contain the information about the adverse reaction and the drug. These data are associated together by the primary ID (primaryid) and case ID (caseid) fields, which uniquely identify the adverse reaction report. The various brand names for the TNF inhibitors IFX, ADA, GOL, CTZ, ETN were identified from the database by querying the active ingredient (prod\_ai) field for INFLIXIMAB, ADALIMUMAB, GOLIMUMAB, CERTOLIZUMAB, and ETANERCEPT. The resulting brand names were then used to query the drug name (drugname) field for all cases pertaining to these drugs. Cases pertaining to pancreatitis were identified by querying the preferred term (pt) field for Pancreatitis. While there could be multiple different drug reactions associated with each case, duplicate entries were dropped by using the unique identifiers primary ID (primaryid) and case ID (caseid).

Statistical analysis

The statistical analysis conducted in this study was based on data mining techniques for disproportionality analysis in pharmacovigilance [20]. These techniques give quantitative measures for detecting associations between a particular drug and a particular adverse drug event. The reporting odds ratio (ROR) compares the odds of pancreatitis events among TNF inhibitor adverse events and the odds of pancreatitis among all other adverse drug events. The Empirical Bayes Geometric Mean (EBGM) compares the percentage of pancreatitis events associated with TNF inhibitors among all adverse events with this percentage assuming that pancreatitis risk is independent of TNF inhibitor use. The information component (IC2) takes the base 2 logarithm of the EBGM. The confidence interval for IC2 takes a Bayesian approach that assumes a prior distribution on the occurrences of the adverse drug events, which makes it more appropriate when the number of observations is relatively small [20]. These statistics are computed based off of an incidence contingency table as displayed in Table 1.

**Table 1:** The 2 x 2 contingency table used for the computation of disproportionality metrics AE: adverse event; TNF: tumor necrosis factor a is the number of adverse drug event reports that report pancreatitis associated with TNF inhibitor use, b is the number of reports that report other AEs associated with TNF inhibitor use, c is the number of reports that report pancreatitis associated with other drugs, and d is the number of other AEs (besides pancreatitis) associated with other drugs (besides TNF inhibitors).

	AE of interest	All other AEs	Total
Drug of interest	a	b	a+b
All other drugs	c	d	c+d
Total	a+c	b+d	N = a+b+c+d

## Results

Baseline characteristics

Our study included data from the fourth quarter of 2012 to the second quarter of 2024. Among the data, a total of 1,549,262 adverse event reports associated with the TNF inhibitors IFX, ADA, GOL, CTZ, and ETN were identified. Among these reports, the number of cases associated with females (n = 998,044) was over 2.5 times higher than the number of cases associated with males (n = 396,485). This is consistent with the well-known fact that there is generally a higher prevalence of autoimmune diseases among women compared to men [21]. When stratified by age, the adverse event reports were predominantly from the adult population, which may be due to a combination of autoimmune disease prevalence and susceptibility to drug adverse effects in this population. Full demographic details are given in Table 2.

**Table 2:** Baseline characteristics of the demographic reporting adverse effects of tumor necrosis factor (TNF) inhibitors

Drug	Total	Sex			Age (years)				
		Female	Male	Unknown sex	<18	18-24	25-64	≥65	Unknown age
Infliximab	245,865	111,476	60,117	74,272	14,140	8,496	84,406	21,170	117,653
Adalimumab	658,932	422,397	199,322	37,213	12,814	17,453	257,969	82,617	288,079
Golimumab	94,119	65,440	18,448	10,231	462	1,092	42,417	14,453	35,695
Certolizumab	118,411	89,610	20,184	8,617	681	1,765	46,638	9,740	59,587
Etanercept	431,935	309,121	98,414	24,400	9,820	5,463	216,427	86,805	113,420
Total	1,549,262	998,044	396,485	154,733	37,917	34,269	647,857	214,785	614,434

Aggregated disproportionality analysis

We first investigated the association between TNF inhibitor use and acute pancreatitis across the entire population and duration of study. Among the TNF inhibitors, we found that IFX had the highest risk for pancreatitis across all measures of disproportionality, with an ROR of 2.13 (95% CI, 2.02, 2.24). For the other four TNF inhibitors, ADA (ROR = 1.04; 95% CI, 0.99, 1.09) and GOL (ROR = 1.11; 95% CI, 0.99, 1.24) exhibited no statistically significant association with pancreatitis, and CTZ (ROR = 0.89; 95% CI, 0.80, 1.00) and ETN (ROR = 0.50; 95% CI, 0.46, 0.54) exhibited a negative association with pancreatitis. The full set of statistics is displayed in Table 3.

**Table 3:** Disproportionality analysis of pancreatitis risk with TNF inhibitor use TNF: tumor necrosis factor; ROR: reporting odds ratio; PRR: proportional reporting ratio; EBGM: empirical Bayes geometric mean; IC2: information component a denotes TNF inhibitor use and pancreatitis, b denotes TNF inhibitor use and no pancreatitis, c denotes other drug use and pancreatitis, d denotes other drug use and no pancreatitis.

Drug	a	b	c	d	ROR (95% CI)	PRR (95% CI)	IC2 (95% CI)	EBGM
Infliximab	1,516	234,307	51,643	16,995,267	2.13 (2.02, 2.24)	2.12 (2.02, 2.23)	1.06 (0.99, 1.14)	2.09
Adalimumab	2,073	647,929	51,086	16,581,645	1.04 (0.99, 1.09)	1.04 (0.99, 1.09)	0.05 (-0.01, 0.12)	1.04
Golimumab	308	89,796	52,851	17,139,778	1.11 (0.99, 1.24)	1.11 (0.99, 1.24)	0.15 (-0.01, 0.32)	1.11
Certolizumab	315	114,083	52,844	17,115,491	0.89 (0.80, 1.00)	0.89 (0.80, 1.00)	-0.16 (-0.32, 0.00)	0.9
Etanercept	656	421,717	52,503	16,807,857	0.50 (0.46, 0.54)	0.50 (0.46, 0.54)	-0.98 (-1.10, -0.87)	0.5

The finding that CTZ and ETN had a negative association with pancreatitis may be partially explained by the fact that some TNF inhibitors, including ETN [12,22] and CTZ [23,24], have shown to have protective function against pancreatitis.

Temporal trends of pancreatitis risk with TNF inhibitor use

**Figure 1:**
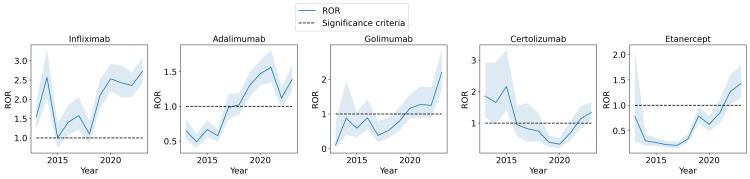
ROR of the association between pancreatitis and TNF inhibitors ROR: reporting odds ratio; TNF: tumor necrosis factor When plotted across years from 2013 to 2023, there is a clear increasing trend in many TNF inhibitors.

Next, we considered the same disproportionality analysis, but stratified by year, to investigate the trends of the pancreatitis risk with TNF inhibitors over time. Figure 1 illustrates the ROR of the association between pancreatitis risk and TNF inhibitor use along with the 95% confidence interval for the ROR statistic as a trend over the years included in the study. A notable increasing trend in the ROR can be identified for almost all five of the TNF inhibitors. In particular, in the most recent full year of 2023, the ROR is 2.72 (95% CI, 2.41, 3.08) for IFX, 1.39 (95% CI, 1.21, 1.59) for ADA, 2.20 (95% CI, 1.70, 2.85) for GOL, 1.34 (95% CI 1.08, 1.67) for CTZ, and 1.43 (95% CI, 1.14, 1.79) for ETN. These all indicate a statistically significant increase in the ROR compared to the results in Table 3 when all of the years are aggregated together. Furthermore, all of the TNF inhibitors exhibit a positive association with pancreatitis in this year, i.e., a 95% confidence interval that lies above 1.

To control for possible confounding factors in the results of the analysis displayed in Figure 1, various stratified analyses that control for prominent confounding factors are conducted in the following sections.

Stratified analysis by sex and age

**Figure 2:**
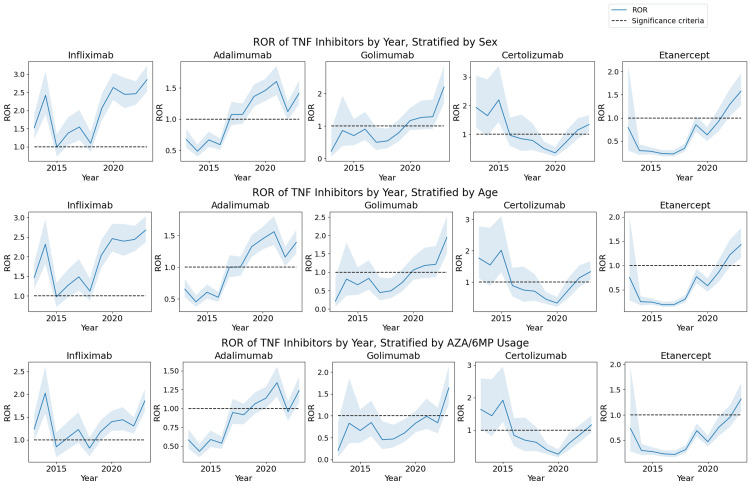
ROR of the association between pancreatitis and TNF inhibitors ROR: reporting odds ratio; TNF: tumor necrosis factor; AZA: azathioprine; 6MP: 6-mercaptopurine The increasing trend in the pancreatitis risk with TNF inhibitors persists after stratification by sex, age, and AZA/6MP usage.

Sex and age are two basic factors to consider as confounding variables. Indeed, there are clear differences between the effect of autoimmune diseases on females versus males [21], so controlling for these factors is critical. In fact, when stratified by sex, we observed that females generally have a statistically higher ROR compared to males for IFX, ADA, GOL, and ETN. For example, the ROR for the association between pancreatitis risk and IFX was 2.55 (95% CI, 2.37, 2.74) in females versus 1.97 in males (95% CI, 1.79, 2.17). Furthermore, GOL exhibited a particularly large gap with ROR = 1.24 (95% CI, 1.08, 1.42) in females versus ROR = 0.67 (95% CI, 0.50, 0.90) in males. The statistics for females and males can be combined by the Mantel-Haenszel method into a single ROR statistic, which is displayed in the first row of Figure 2 by year. A similar increasing trend as observed in Figure 1 is present in Figure 2.

Similarly, the data can be stratified by age group, according to the groups considered in Table 2, and combined via the Mantel-Haenszel method. Under this stratification, it was observed that pediatric patients under the age of 18 generally have a statistically higher ROR compared to other age groups. For example, this population had ROR = 2.19 (95% CI, 1.82, 2.63) for IFX while the age group with the next highest ROR had ROR = 1.62 (95% CI, 1.49, 1.77). Similarly, for GOL, pediatric patients had ROR = 5.42 (95% CI, 2.89, 10.15) while the age group with the next highest ROR had ROR = 2.21 (95% CI, 1.14, 4.27). The overall combined ROR after stratification by age plotted by year is illustrated in the second row of Figure 2. Again, the result ROR trend is similar to the trends observed in Figure 1.

Effects of combination therapy with azathioprine

Next, we conducted a study to isolate the effect of azathioprine and mercaptopurine (AZA/6MP), which is commonly prescribed together with TNF inhibitors, especially IFX and ADA, in combination therapies [15,16]; it has been known for its association with drug-induced pancreatitis [17,18].

After stratification, the overall aggregate disproportionality analysis for IFX exhibited a significantly lower ROR. In particular, the population of patients not using AZA/6MP had ROR = 1.46 (95% CI, 1.37, 1.56) while the population of patients using AZA/6MP had ROR = 1.30 (95% CI, 1.18, 1.43). Thus, a significant part of the association between IFX and pancreatitis was explained by the confounding factor of concurrent AZA/6MP use. After controlling for this stratification, the combined ROR using the Mantel-Haenszel method was 1.40 (95% CI, 1.33, 1.48). It can be observed that although the association with pancreatitis weakens after this stratification, an independent component of pancreatitis risk still exists with IFX use.

Even after controlling for the use of AZA/6MP, an increasing trend in the ROR of IFX and the other TNF inhibitors continues to exist, as illustrated in the third row of Figure 2. In particular, in the year 2023, the RORs of the pancreatitis risk with TNF inhibitors are given by ROR = 1.86 (95% CI, 1.62, 2.12) for IFX, ROR = 1.24 (95% CI, 1.08, 1.42) for ADA, ROR = 1.64 (95% CI, 1.26, 2.12) for GOL, ROR = 1.16 (95% CI, 0.93, 1.45) for CTZ, and ROR = 1.32 (95% CI, 1.06, 1.63) for ETN.

Stratification by indication of use

**Figure 3:**
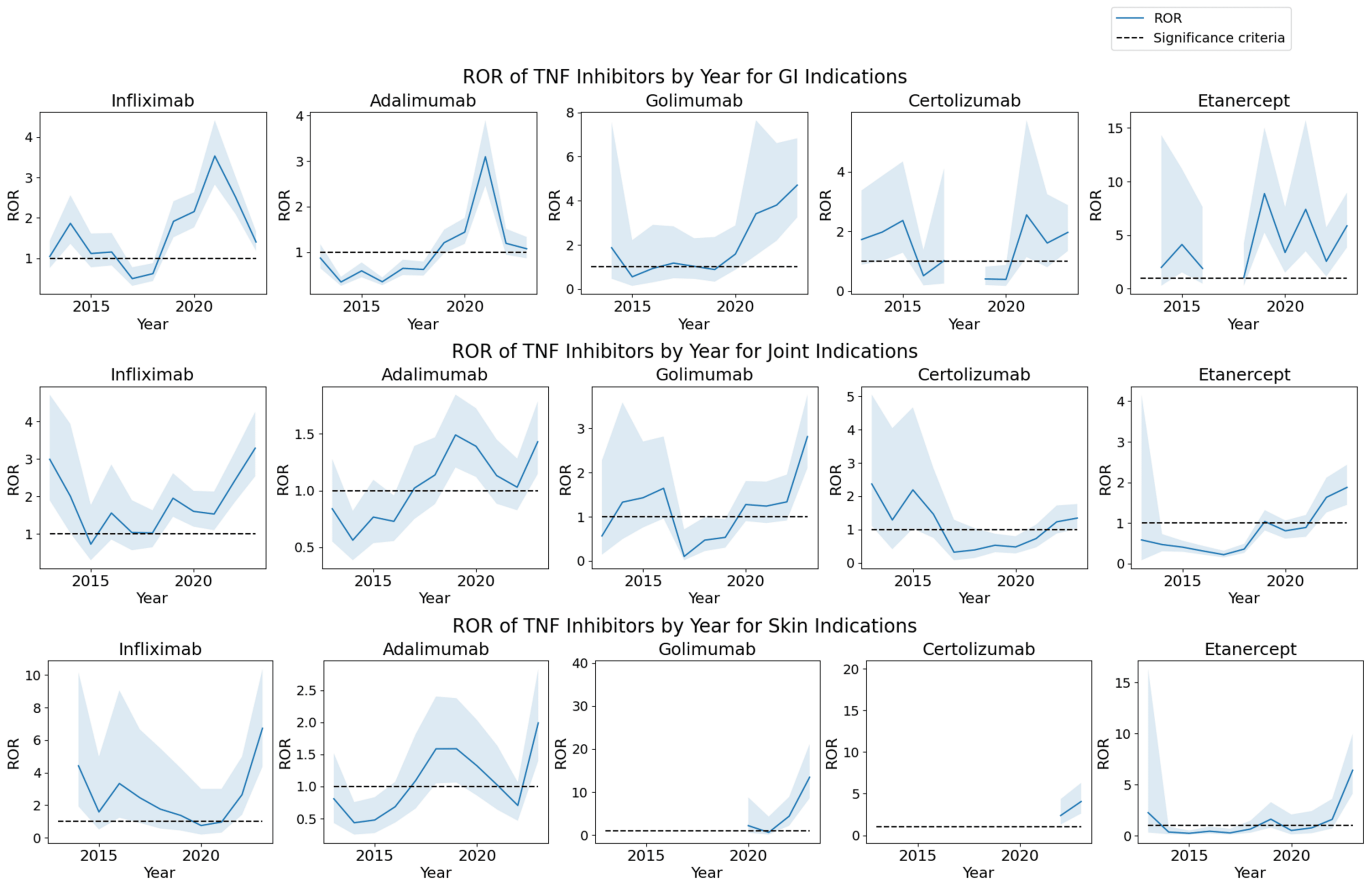
ROR of the association between pancreatitis and TNF inhibitors ROR: reporting odds ratio; TNF: tumor necrosis factor The increasing trend in the pancreatitis risk of TNF inhibitors persists after stratification by the indication for TNF inhibitor treatment.

Finally, we conducted an analysis that stratified the population studied by the indication of treatment by TNF inhibitors. Three of the major categories of indications for TNF inhibitors included gastrointestinal (GI) indications such as inflammatory bowel disease and irritable bowel syndrome, joint indications such as rheumatoid arthritis and ankylosing spondylitis, and skin indications such as dermatitis and psoriasis. These categories of indication were identified by the first author by searching for relevant keywords such as "bowel", "arthritis", "dermatitis", and a variety of other keywords. A disproportionality analysis was then conducted on each of these subsets of cases. The results for each of these categories of indications, plotted by year, are displayed in Figure 3. It can be observed that the increasing trend still exists in each of the categories of indication. Furthermore, the ROR values are significantly elevated in many cases, compared to the ROR computed from the overall population of the study. This indicates that reports of pancreatitis adverse events are distributed more among these indications of TNF inhibitor therapy.

## Discussion

We studied the association between acute pancreatitis and TNF inhibitors IFX, ADA, GOL, CTZ, and ETN. We conducted a disproportionality analysis using the FAERS database, analyzing over a decade’s worth of data. Our analysis revealed that across the entire data, IFX had the largest association with pancreatitis (ROR = 2.13; 95% CI, 2.02, 2.24). Furthermore, all of the TNF inhibitors exhibited an increasing trend when stratified by year, with a significantly elevated ROR seen across all of the TNF inhibitors in the year 2023. Nearly identical conclusions were drawn when the data were stratified according to sex or age and combined via the Mantel-Haenszel method (Figure 2). When the data were stratified by the concurrent AZA/6MP use, the ROR was significantly decreased for IFX, which indicates that AZA/6MP explains a significant part of the pancreatitis risk associated with IFX. However, even after this stratification, the pancreatitis risk of IFX was still significant, and furthermore, still exhibited an increasing trend (Figure 2). Finally, the increasing trend was present even when stratifying the dataset by the indication for TNF inhibitor treatment including GI, joint, and skin indications, and furthermore, the ROR was further significantly increased (Figure 3).

An interesting aspect of the relationship between TNF inhibitors and pancreatitis is that in animal models, it has in fact been shown that TNF inhibitors may be an effective therapy for pancreatitis [25,26]. Our analysis also indicated that TNF inhibitors can be negatively associated with pancreatitis, for example, for ETN. Therefore, we believe that an important direction for future research is to study what makes TNF inhibitors protective against pancreatitis in certain settings and what makes TNF inhibitors risk factors for pancreatitis in others.

Numerous other pharmacovigilance studies of the adverse events of TNF inhibitors have been conducted [27-30]. Some of the specific adverse events studied include glioblastoma [28] and hypoglycemia [30]. However, none of these works studied the risk of pancreatitis associated with TNF inhibitor use, which is a particularly important adverse effect as pointed out by a large number of case reports [7-13].

This study has several limitations that could be improved upon in future studies on TNF inhibitor-associated pancreatitis. While we have attempted to control for various confounding variables that may exist such as age, sex, and concomitant AZA/6MP use, there may still be several important variables that we were unable to account for in this study. In clinical reports on drug-induced acute pancreatitis associated with TNF inhibitor use, it is common to also report other risk factors for acute pancreatitis such as gallstones, hypertriglyceridemia, alcohol use, and serum immunoglobulin assays such as IgG4. These additional tests are useful for excluding cases of acute pancreatitis that can be attributed to causes other than TNF inhibitors, such as gallstone pancreatitis, pancreatitis secondary to hypertriglyceridemia, alcoholic pancreatitis, and autoimmune pancreatitis. Therefore, a further in-depth study of this adverse drug relationship should account for these evaluations as well. Finally, the data collected in the FAERS database itself may contain certain selection biases due to the database being built off of spontaneous adverse event reports.

## Conclusions

Following up on numerous case reports reporting drug-induced acute pancreatitis associated with TNF inhibitors, we conducted a systematic investigation of the adverse drug reaction relationship between the TNF inhibitors IFX, ADA, GOL, CTZ, and ETN and pancreatitis. Our results revealed a statistically significant association between pancreatitis risk and usage of IFX, and a prominent increasing trend in the pancreatitis risk with IFX, ADA, GOL, and ETN. Similar trends were observed even after controlling for sex, age, concomitant AZA/6MP use, and indication for treatment. Together, our findings highlight the necessity for careful monitoring of pancreatitis in patients undergoing TNF inhibitor therapy.
